# Preparation and Thermal Properties of Molecular-Bridged Expanded Graphite/Polyethylene Glycol Composite Phase Change Materials for Building Energy Conservation

**DOI:** 10.3390/ma11050818

**Published:** 2018-05-16

**Authors:** Dong Zhang, Meizhu Chen, Quantao Liu, Jiuming Wan, Jinxuan Hu

**Affiliations:** State Key Laboratory of Silicate Materials for Architectures, Wuhan University of Technology, Wuhan 430070, China; pytmac@whut.edu.cn (D.Z.); liuqt@whut.edu.cn (Q.L.); wanjm@whut.edu.cn (J.W.); hujinxuan221@whut.edu.cn (J.H.)

**Keywords:** expanded graphite, polyethylene glycol, phase change materials, titanate coupling agent, molecular bridge, building envelopes, thermal property, building energy conservation

## Abstract

Using phase change materials (PCMs) in building envelopes became a reliable method to improve indoor comfort and reduce buildings’ energy consumption. This research developed molecular-bridged expanded graphite (EG)/polyethylene glycol (PEG) composite PCMs (m-EPs) to conserve energy in buildings. The m-EPs were prepared through a vacuum absorption technique, and a titanate coupling agent was used to build a molecular bridge between EG and PEG. SEM, mercury intrusion porosimetry (MIP), the leakage test, microcalorimetry, X-ray photoelectron spectroscopy (XPS), and Fourier transform infrared spectroscopy (FT-IR) were conducted to characterize the morphology, pore structure, absorbability, and modifying effects of the m-EPs. The phase change temperature, latent heat, thermal stability, and thermal conductivity of the m-EPs were determined by a differential scanning calorimeter (DSC), TGA, and a thermal constants analyzer. Results showed that the maximum mass ratio of PEG to EG without leakage was 1:7, and a stable connection was established in the m-EPs after modification. Compared with the unmodified EPs, the supercooling degree of the m-EPs reduced by about 3 °C, but the latent heats and initial decomposition temperatures increased by approximately 10% and 20 °C, respectively, which indicated an improvement in the thermal energy storage efficiency. The thermal conductivities of the m-EPs were 10 times higher than those of the pristine PEGs, which ensured a rapid responding to building temperature fluctuations.

## 1. Introduction

The rapid development of human civilization has led to a rising demand for energy. Statistics show that the global energy consumption reached 6.607 × 10^14^ MJ so far, and more than three-quarters of them are conventional fossil fuels (such as coal, petroleum oil, and natural gas) [1,2]. The extensive use of non-renewable energy leads to severe resource scarcity and environmental pollution problems [3]. On the other hand, the residential and commercial buildings consume almost 40% of the world’s total energy usage for heating, ventilating, and air conditioning, which make it the leading energy consuming sector [4]. Therefore, exploiting green and energy-efficient buildings would be of benefit to the solution of energy and environment challenges facing the world. Applying phase change materials (PCMs) in building envelopes (such as wallboard [5], concrete [6], and insulation materials [7]) is a promising approach to decrease the energy consumption in buildings. The PCMs in building envelopes can spontaneously absorb thermal energy during hot daytime, and release thermal energy when the surrounding temperature dropped at nighttime. The phase transition of PCMs is a spontaneous process, and the phase transition temperature is constant. Therefore, using PCM-enhanced building envelopes to regulate temperature is an ideal way of improving the indoor comfort and conserving building energy. 

Based on the phase transition modes, PCMs can be classified into different types such as solid–solid, solid–liquid, solid–gas, and liquid–gas [8]. Up to now, the solid–liquid type PCMs with the merits of volume stability, proper phase change temperatures, and lower cost have become the most widespread PCMs in building thermal management [9]. The solid–liquid PCMs can be categorized into inorganic, organic, and eutectic mixture according to their chemical compositions. The inorganic PCMs refer to various salt hydrates that have high thermal energy storage density, high thermal conductivity, and low cost. However, these materials are subject to many constraints such as corrosion, supercooling, and segregation during their service [8]. Comparatively, the organic PCMs, such as paraffins, carbohydrates, and derived lipids exhibit broader prospects due to their favorable chemical stability, heat of fusion, and resistances to supercooling and phase separation. Despite the flaws of low-thermal conductivity and liquid leakage, the organic PCMs are still the most widely used [10]. As for the eutectic mixture, it refers to a combination of two or more either organic or inorganic compounds, or a mixture of both [11]. The melting point of eutectics can be tailored to any desired temperature. However, the advantage of custom-tailoring also results in the high cost of eutectics, which is commonly two or three times greater than the organic or inorganic compounds.

As the most favorable PCMs in building envelopes, a host of attempts have been proposed to overcome the shortcomings of organic PCMs. Finned tubes [12], heat-conducting fillers [13], and a metal/graphite matrix [14] have been applied to enhance the thermal conductivity of organic PCMs. Meanwhile, the encapsulation approaches such as the in situ polymerization method [15], complex coacervation method [16], sol–gel method [17,18], and solvent extraction/evaporation method [19] were developed to fabricate form-stable PCMs (FSPCM). Among all of these performance-enhancing methods of organic PCMs, using expanded graphite (EG) as a matrix to absorb solid–liquid PCMs is an ideal way to enhance the heat transfer rate as well as prevent leakage. EG is a porous carbonaceous material with favorable absorbability, thermal conductivity, and chemical stability, and its applications on the encapsulation of PCMs has become a research focus in recent years. Scholars have conducted constructive studies on the fabrication and application of EG-based FSPCMs. Zhang et al. [20] and Sari et al. [21] verified the feasibility of applying EG as a heat transfer enhancer and shape stabilizer for paraffin PCMs. Xia et al. [22], Zeng et al. [23], Wang et al. [24], and Ling et al. [25] have separately applied various organic PCMs as functional components, and performed a series of tests on the morphologies, absorptive capacities, thermal conductivities, phase change temperatures, and enthalpies of the EG-based FSPCMs. Zhang et al. [26], Li et al. [27], and He et al. [28] incorporated different EG-based FSPCMs into cement mortars and evaluated their thermal energy storage performances. The results indicated that EG-based FSPCMs could reduce the indoor temperature variation and energy consumption of buildings.

Although the EG-based FSPCMs have been successfully applied in building envelopes, the liquid leakage and performance degradation are still inevitable to a certain extent, because the PCMs are bonded with the EG matrix only through weak physical connections (capillarity and van der Waals force). Meanwhile, few attempts have been made to enhance the interaction between EG and PCMs. Therefore, the objective of this research was to fabricate a novel EG-based FSPCM with strong chemical bonding. EG matrices with different particle sizes and pore structures were used to absorb polyethylene glycol (PEG). The optimal EG matrix for PEG absorption was determined by detecting the absorbability and weight loss of EG/PEG composite PCMs (EPs) under a high temperature. A titanate coupling agent KR-38S was employed to build a molecular bridge between an EG matrix and PEG. The modified EG matrix (m-EG) was prepared and mixed with five different PEGs to fabricate molecular-bridged EPs (m-EPs). Thermal properties, including the phase change temperature, supercooling degree, latent heat, heat transfer rate, and thermal reliability of m-EPs were characterized to demonstrate their performance improvement in this research. 

## 2. Materials and Methods

### 2.1. Materials

Polyethylene glycols (PEGs, 98%, chemical pure) with different relative molecular mass (800, 1000, 1500, 2000, and 3000, named as PEG_800_, PEG_1000_, PEG_1500_, PEG_2000_, and PEG_3000_, respectively) were supplied by Sinopharm Chemical Reagent Co., Ltd. (Wuhan, China). An isopropyl tri-(dioctylpyrophosphate) titanate coupling agent (C_51_H_112_O_22_P_6_Ti, KR-38S) was obtained from Kenrich Petrochemicals Inc. (New York, NY, USA). Expanded graphite (EG) with different particle sizes (45 μm, 75 μm, 125 μm, 180 μm, and 300 μm, named as EG_45_, EG_75_, EG_125_, EG_180_, and EG_300_, respectively) were purchased from Qingdao Graphite Co., Ltd. (Qingdao, China). All of the materials were used as received without any further purification. The basic properties of PEGs and EGs were tested and demonstrated in Table 1 and Table 2, respectively.

### 2.2. Selection of EG Matrix for PEG Absorption

**Figure 1:**
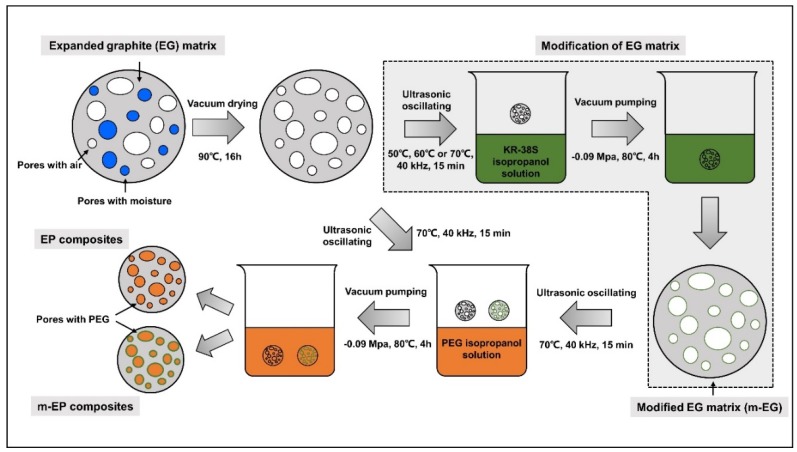
Schematic for the preparation of molecular-bridged EG/PEG composite phase change materials (m-EPs).

Exploratory experiments were conducted to investigate the influence of PEGs molecular mass on the absorption capacity of EG. Results showed that no connection existed between the two. Therefore, PEG_2000_ was selected as a PCM in this section to explore the optimal EG particle size for PEG absorption and the maximum absorbability. The EG/PEG composite PCMs (EPs) were fabricated in the following steps. First, the EG matrix was desiccated in a vacuum oven at 90 °C in order to weigh it accurately. Afterwards, the weighted EG matrix was mixed with PEG_2000_ isopropanol solution proportionally by an ultrasonic oscillation at 70 °C for 15 min; the oscillating frequency was 40 kHz. Finally, the mixtures were treated in a vacuum pump at 80 °C for 4 h, in order to vaporize the isopropanol solvents and absorb the melted PEG_2000_. The schematic for the preparation of EPs was shown in Figure 1.

The optimal particle size of EG for the absorption of PCMs was determined by a series of experiments. First, liquid leakage tests were conducted by spreading the EP samples on filter papers uniformly and heating them in a vacuum oven at 85 °C for 1 h. The weight loss and mass ratio of each sample were considered as the indicators to select an optimal EG matrix with favorable thermal stability and absorptivity. Subsequently, mercury intrusion porosimetry (MIP, AutoPore IV 9510, Micromeritics Instrument Corp., Norcross, GA, USA) and a scanning electron microscope (SEM, Quanta 450 FEG, FEI, Hillsboro, OR, USA) were employed to characterize the pore structure and morphology of each EG matrix and the corresponding EP. Finally, the optimal EG matrix was determined based on the aforementioned tests results.

### 2.3. Preparation of Modifid EG (m-EG) and Molecular-Bridged EP (m-EP)

The absorption and compatibility between an EG matrix and PCMs depend mainly on the functional groups on the EG surface, and it was critical to modify the EG according to the PCM category and working condition of the FSPCM. KR-38S is a titanate coupling agent with high coupling efficiency on polymers and inorganic fillers. The modification principle of KR-38S on the EG matrix in this research can be described as follows: the alkoxy groups of KR-38S react with the hydroxyl groups of the EG matrix to produce a monomolecular layer on the EG surface. When the PEG was mixed with the modified EG (m-EG), a transesterification reaction between KR-38S and the terminal hydroxyl groups of PEG (which have similar properties as alcoholic hydroxyls) could occur on the surface of m-EG; consequently, a strong combination between m-EG and PEG was established by using KR-38S as the molecular bridge.

The schematic of EG matrix modification was also shown in Figure 1. The EG matrix was dried in a vacuum oven at 90 °C for 16 h to remove moisture. Then, the isopropanol solutions with different KR-38S dosages (1 wt %, 2 wt %, 3 wt %, and 4 wt % of EG) were prepared and mixed with the EG matrix. The mixing process was performed by ultrasonic oscillation at three different temperatures (50 °C, 60 °C, and 70 °C) to simulate different modifying conditions. The time and frequency of modifications were 15 min and 40 kHz, respectively. When the ultrasonic treatment was complete, the isopropanol solvents were vaporized in a vacuum pump at 80 °C for 4 h, and the modified EG matrix (m-EG) was fabricated. Microcalorimetry (C80, Setaram, Caluire, France) and X-ray photoelectron spectroscopy (XPS, ESCALAB 250Xi, Thermo Fisher Scientific, Waltham, MA, USA) were used to detect the isothermal calorimetric curve of each reaction and chemical state of the m-EGs, in order to determine the optimal modifying condition.

### 2.4. Characterization of m-EPs

The properties of EPs and m-EPs were characterized in this section. The phase change temperatures and latent heats were investigated by a differential scanning calorimeter (DSC, Pyris1DSC, Perkin Elmer, Waltham, MA, USA). Indium was selected as a reference for instrument calibration. The heating rate was 1 °C/min, and the testing temperature range was 0–70 °C. Fourier transform infrared spectroscopy (FT-IR, Nicolet™ 6700, Thermo Fisher Scientific, Waltham, MA, USA) was employed to manifest the chemical composition of EPs and m-EPs. The scanning range was from 4000 cm^−1^ to 400 cm^−1^ with a 4 cm^−1^ resolution. A thermogravimetric analyzer (TGA, STA449F3 Jupiter, NETZSCH, Bavaria, Germany) was used to compare the thermal stabilities of EPs and m-EPs. The measurements were conducted from the ambient temperature to 700 °C at a heating rate of 10 °C/min, the experimental atmosphere was N_2_, and the flow rate was 100 mL/min. Thermal conductivities were measured by using a thermal constants analyzer (TPS 2500S, Hot Disk, Goteborg, Sweden). Samples were prepared by a dry-pressing process with a cylindrical mold of 45 mm in diameter and 15 mm in height. The packing density of all of the samples was 1.13 ± 0.02 g/cm^3^, which was consistent with that of the pristine PEGs, in order to avoid the influence of packing density on thermal conductivity. 

## 3. Results and Discussion

### 3.1. Preparetion of m-EPs

#### 3.1.1. Selection of EG Matrix

**Figure 2:**
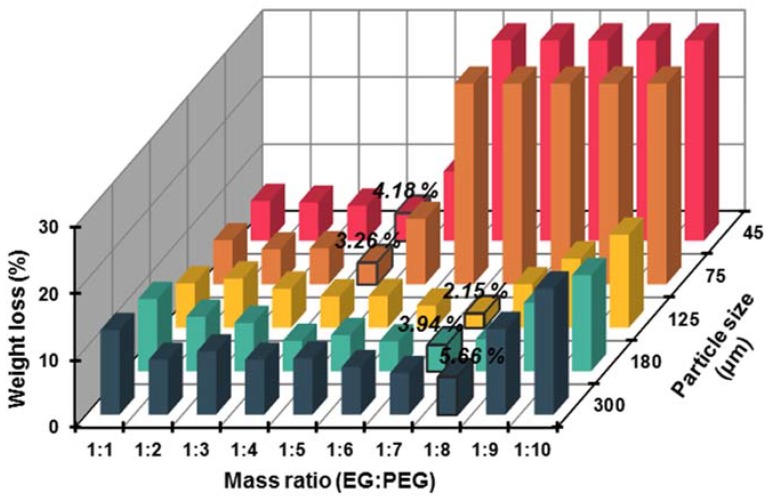
Weight loss of different EG/PEG composite phase change materials (EPs) after heat treatment.

Latent heat is critical for the application of PCMs; typically, a higher latent heat is beneficial for an efficient and economical utilization of building envelopes. Therefore, it is vital to incorporate more PEGs in the limited pore volumes of EG without degrading its performance. Five kinds of EG matrices were selected as the supporting materials to absorb PEG_2000_, and the mass ratios of EG and PEG_2000_ were 1:1, 1:2, 1:3, 1:4, 1:5, 1:6, 1:7, 1:8, 1:9, and 1:10, respectively. The leakage tests were performed to determine the optimal supporting material and the maximum absorbability. As presented in Figure 2, the weight loss percentages of five kinds EG matrices exhibited a similar trend that decreased initially and then increased, which indicated that each EG matrix had a certain absorbability. Specifically, the EG_300_ matrix exhibited the minimum weight loss percentage (5.66 wt % of EP) when the EG/PEG mass ratio was 1:8, while both the minimum weight loss percentages of EG_180_ and EG_125_ (3.94 wt % and 2.15 wt % of the corresponding EPs, respectively) were achieved when the EG/PEG mass ratio was 1:7. This phenomenon might be because EG_300_ possessed abundant pore volume compared with EG_180_ and EG_125_ (as seen in Table 2). Meanwhile, the macropores and mesopores in EG_300_ were more suitable for the adsorption of large molecules. Despite a higher absorbability, the weight losses of EG_300_ in each mass ratio were also higher in comparison with EG_180_ and EG_125_. Therefore, the packaging stability of the EG_300_ matrix was considered inferior to that of EG_180_ and EG_125_. Similarly, EG_125_ was a better supporting material than EG_180_ in this research. As for EG_75_ and EG_45_, their maximum EG/PEG mass ratios were both 1:4, and the weight losses increased dramatically when the mass ratios exceeded this threshold. This phenomenon can be explained by the limited amount of absorptive macropores and mesopores in EG_75_ and EG_45_ hindering the absorption of PEG. Hence, the EG_125_ matrix, which had the maximum EG/PEG mass ratio of 1:7, was considered as the optimal supporting material for PEG in this research.

The pore structure parameters and micromorphology of various EGs and the corresponding EPs with the maximum PEG absorption capacities are shown in Table 3 and Table 4, respectively. As presented in Table 2, the specific surface areas of the EG matrices decreased as their particle sizes increased; however, the pore volumes and average pore radii exhibited the opposite tendencies. This could be because the higher expansion ratio of the large-sized EG particles resulted in an increase of macropores and mesopores for absorption, as well as a reduction in the amount of micropores that have large specific surface areas. When the maximum absorption capacities were achieved, the specific surface area, pore volume, and average pore radius of various EGs showed significant reductions, as illustrated in Table 3. These phenomena proved that the PEG was absorbed in the framework of the EG matrix. Furthermore, the leakage tests demonstrated that the maximum PEG absorption capacities increased from 1:4 to 1:8 as the EG particle size increased, and this trend was ascribed to the quantity variance of macropores. 

The SEM images showed that all of the EG matrices had the worm-like structures, which were favorable to expanding the surface areas and enhancing the absorption capacities. It also can be noted that with the increase in EG particle size, the folding-type macropores, which ensured the great surface tension and capillary force of the EG matrix, were increased. These results were consistent with the outcomes of the pore structure analyses. As for the EPs, different features were exhibited in their morphologies. The surfaces of EG_45_ and EG_75_ were coated with massive PEG crystal when the mass ratios of EG/PEG were 1:4. On the other hand, when the absorption capacities of EG_125_, EG_180_, and EG_300_ were achieved, the frameworks of these EGs were still distinct, except for a slight amount of PEG crystal on the surface. Considering the variance in maximum absorption capacity, it can be concluded that the absorption of the EG_45_ and EG_75_ matrices to PEG was mainly depended on their extensive surface areas, while the massive macropore structures were responsible for the absorption of EG_125_, EG_180_, and EG_300_. It is also noteworthy that the worm-like structures of EG_180_ and EG_300_ were slightly damaged, owing to their high expansion ratios. These structural defects might be detrimental to the further processing of EPs. Therefore, based on the analyses of pore structures and morphologies, the EG_125_ matrix, which had the maximum PEG absorption capacity of 1:7, was determined to be the optimal supporting material in this research. 

#### 3.1.2. Modification of EG Matrix

**Figure 3:**
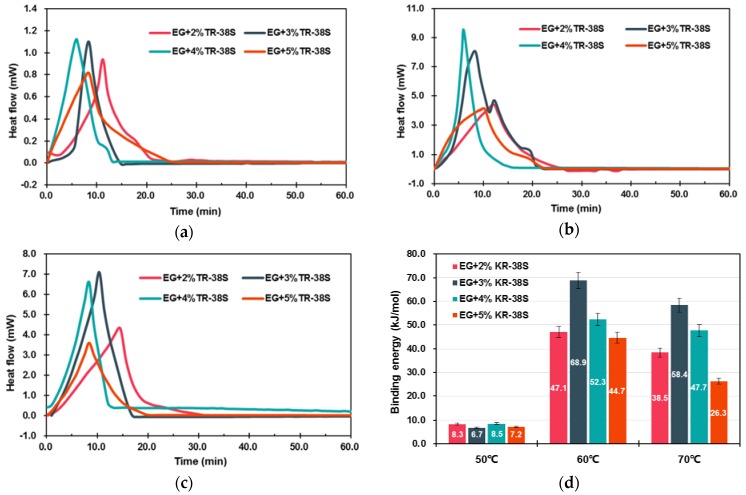
Calorimetric curves and binding energy values for the preparation of modified EGs (m-EGs). (**a**) calorimetric curves at 50 °C; (**b**) calorimetric curves at 60 °C; (**c**) calorimetric curves at 70 °C; (**d**) binding energy of reactions.

The occurrence of chemical and physical reactions is always accompanied by the release or absorption of heat. Analyzing the heats would be helpful for determining the category and characteristic of the reactions. Figure 3 illustrated the isothermal calorimetric curves and binding energy values of EG-modifying reactions. As shown in the calorimetric curves of Figure 3, the reactions between EG and KR-38S were exothermic, and the shapes as well as the positions of the peaks varied with the KR-38S dosage and reaction temperature. These results indicated that the modifier was absorbed on the surface of the EG, and the absorption efficiencies were dependent on the modification conditions. To be specific, the binding energy values of the modifications at 50 °C were less than 10 kJ/mol, indicating that the coupling agent molecules were unactivated, and the physisorption (less than 40 kJ/mol of binding energy) that was caused by the van der Waals force was dominant [29]. When the temperature increased to 60 °C, the binding energy of each sample exhibited a significant increase. It can be inferred that a chemisorption between the EG surface and the coupling agent molecules might have occurred. Furthermore, the binding energy at 60 °C initially increased, and decreased subsequently as the KR-38S dosage increased. This phenomenon can be explained by the modification being optimized when a monomolecular layer was created on the surface of the EG. Adding an excessive coupling agent that was hampered the chemical reaction between KR-38S and EG consequently resulted in the reduction of binding energy. As for the modifications at 70 °C, the binding energy values demonstrated the same tendencies, but lower values in comparison with those at 60 °C. The reason was that the modification temperature had exceeded the optimal activation temperature of KR-38S, and the chemisorptions were hindered. Therefore, it could be concluded from this section that the chemisorption could occur between the coupling agent and the EG matrix, and the optimal modifying condition was obtained by adding 3 wt % of KR-38S at 60 °C. The chemisorptions enhanced the connection between the EG and PEGs, which was beneficial for improving the thermal stability and energy storage density of the m-EPs, as well as the energy conservation efficiency of the building envelopes. 

**Figure 4:**
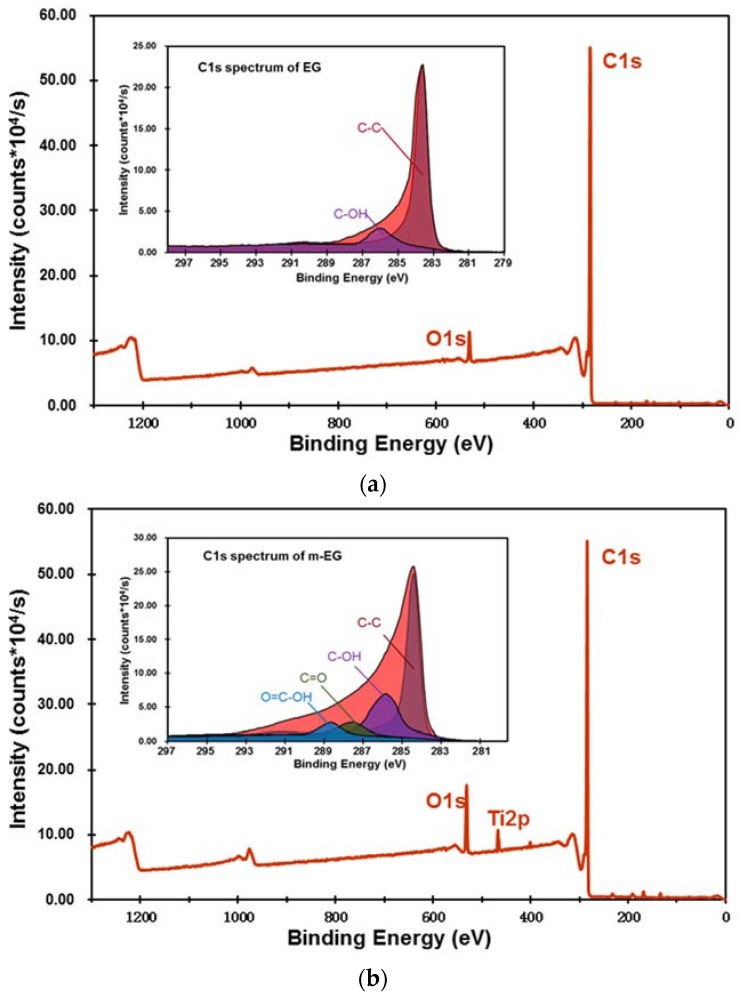
X-ray photoelectron spectroscopy (XPS) spectra of the EG matrix (**a**) and the m-EG matrix (**b**).

The XPS spectra of EG and m-EG are demonstrated in Figure 4. It was noticeable that two characteristic spectral peaks of O1s and C1s were illustrated in the spectrum of the EG matrix. The O1s was the oxidant residual during EG fabrication and adventitious carbon contamination in the atmosphere. Comparing with the EG matrix, the intensity of the O1s peak was obviously higher in the spectrum of the m-EG, and a new characteristic peak at 462.2 eV, corresponding to Ti2p, emerged. The peak table indicated that the oxygen element content in the EG matrix increased from 3.17 at % to 11.21 at % with the modification of KR-38S, and the titanium element was increased from 0.19 at % to 2.71 at %. This indicated that the coupling agent was attached on the surface of the EG matrix. In order to better investigate the modification mechanism, the C1s spectra of the EG and m-EG was deconvoluted by using the Gauss–Lorentz function, and at the same time, the chemical state of the C1s electron was characterized [30]. It was obvious that two dominant peaks with bonding energies of 284.6 eV and 285.5 eV, corresponding to C-C and C-OH, respectively, were shown in the C1s spectrum of the EG. The appearance of a C-OH peak indicated the existence of hydroxyl groups on the EG surface. Compared with EG, the C1s spectrum of m-EG exhibited another two peaks at 287.5 eV and 288.9 eV corresponding to C=O and O=C-OH, respectively. These new peaks were ascribed to the reactions between KR-38S and the EG matrix. The XPS results indicated that a satisfying modification effectiveness on the EG matrix was achieved, which was consistent with the conclusion of the microcalorimetry tests. 

#### 3.1.3. Preparation and Chemical Composition of m-EP

**Figure 5:**
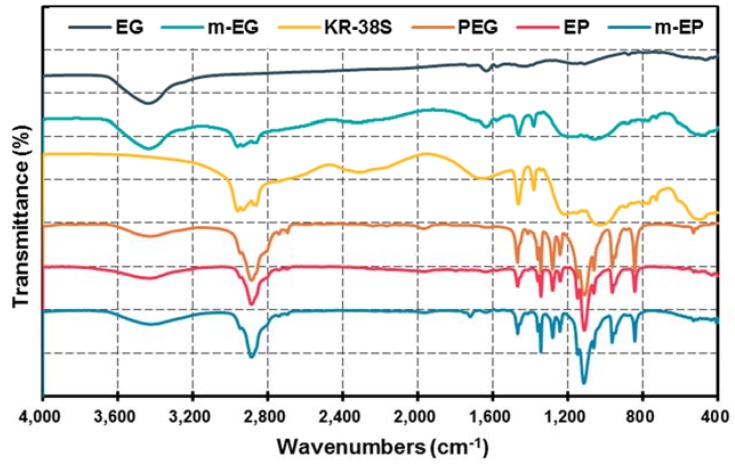
Fourier transform infrared (FT-IR) spectra of EG, the modified EG matrix (m-EG), KR-38S, PEG, EP, and m-EP.

The FT-IR spectra of the EG, m-EG, KR-38S, PEG, EP, and m-EP at the wavenumbers between 4000 cm^−1^ and 400 cm^−1^ were shown in Figure 5. From the spectrum of EG, three peaks at the wavenumbers of 1641 cm^−1^, 1580 cm^−1^, and 1432 cm^−1^, which were caused by the stretching vibration of C=C, the stretching vibration of C=O, and the in-plane bending vibration of -OH, respectively, could be noticed. As for the coupling agent KR-38S, the chief characteristic peaks that can be observed in the spectrum were the symmetrical and asymmetrical stretching vibrations of -CH_3_ at 2958 cm^−1^ and 2875 cm^−1^, respectively. Moreover, the symmetrical and asymmetrical deformation vibrations of -CH_3_ at 1464 cm^−1^ and 1386 cm^−1^ could also be detected. Furthermore, the characteristic peaks at 1036 cm^−1^ and 609 cm^−1^, corresponding to the vibrations of P-O and Ti-O, respectively, were critical for determining the type of coupling agent. When the modification of the EG matrix was performed, the four mentioned characteristic peaks belonging to the stretching and deformation vibrations of KR-38S appeared in the spectrum of m-EG matrix, but the in-plane bending vibration of -OH at 1432 cm^−1^ disappeared. This phenomenon could be because the peak position of the -OH in-plane bending vibration overlapped with the position of the symmetrical and asymmetrical deformation vibrations of -CH_3_.

Comparing the spectra of PEG and EP, it was notable that most of the characteristic peaks appeared in both spectra, except for some slight shifts. To be specific, the characteristic peaks at 845 cm^−1^ and 961 cm^−1^ corresponded to the bending vibrations of the -CH_2_CH_2_O- and -C-O-C- functional groups, respectively. The peaks at 1103 cm^−1^ and 1149 cm^−1^ were the stretching vibration of C-O. The peaks at the wavenumbers of 1242 cm^−1^ and 1281 cm^−1^ were the results of -OH symmetrical and asymmetrical stretching vibrations, respectively. The peaks at 1342 cm^−1^ and 1469 cm^−1^ represented the bending vibrations of the functional groups of -CH_2_ and -OH, respectively. The similar FT-IR spectrum features between the EP and PEG indicated that the reaction between PEG and EG mainly depended on the capillary force and hydrogen bonding, rather than the chemical bonding. As for the m-EP, the characteristic peak at wavenumber of 1802 cm^−1^, representing the symmetrical stretching vibration of anhydride C=O, disappeared. However, a new peak belonging to the stretching vibration of ester C=O appeared at 1781 cm^−1^. These results indicated that a transesterification between KR-38S and PEG might have occurred, and a chemical combination was established in m-EP.

### 3.2. Thermal Properties of m-EPs

#### 3.2.1. Latent Heat and Phase Change Temperature

**Figure 6:**
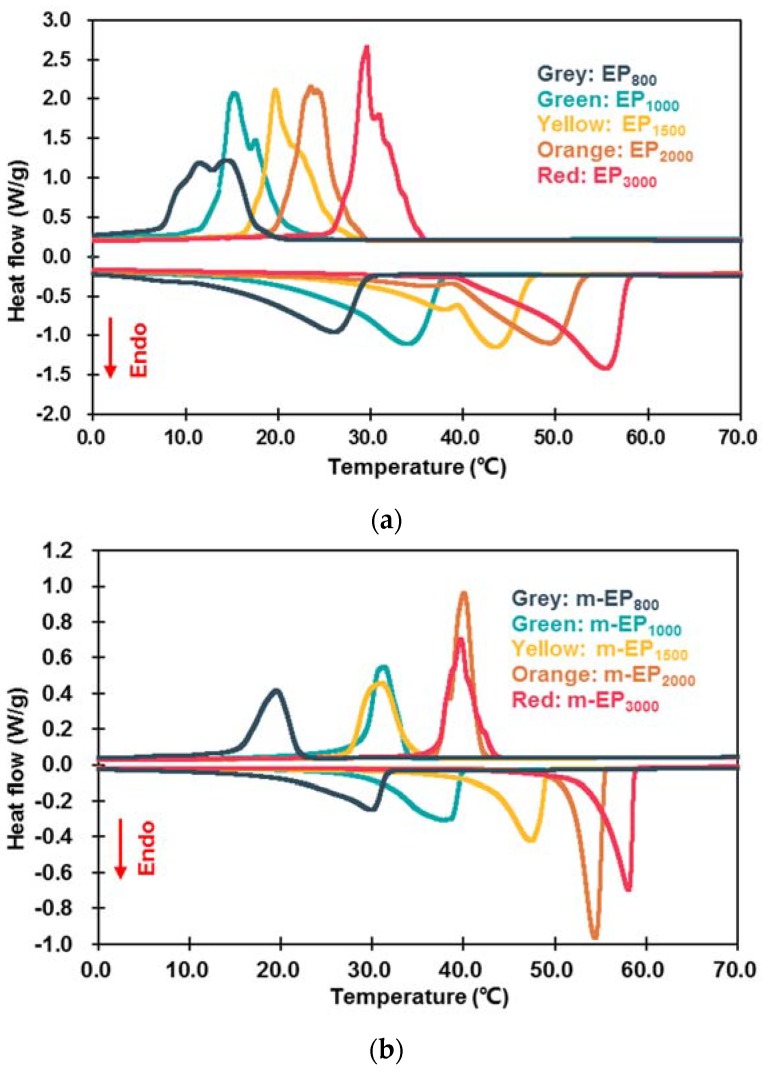
Differential scanning calorimeter (DSC) curves of EPs (**a**) and m-EPs (**b**).

Figure 6 demonstrated the DSC curves of the EPs and m-EPs. Their corresponding thermal properties are summarized in Table 5. As seen in Figure 6, the melting and crystallization temperatures of the EPs and m-EPs increased with the increase in the molecular mass of PEG. This feature was consistent with the trend of pristine PEG, indicating the successful incorporation of PEGs into the EG matrix. Meanwhile, the DSC curves of the m-EPs were smoother, and the endothermic/exothermic peaks were narrower in comparison to those of the EPs. This result indicated that applying a m-EG was beneficial for the stabilization of the FSPCM phase change process in the building envelopes. Specifically, the EPs exhibited lower *T_onset-m_* and *T_onset-c_* values compared with those of the PEGs, because the crystallization-promoting effects of the EG surface accelerated the crystallization of PEGs [31]. Furthermore, the *T_onset-m_* and *T_onset-c_* of the m-EPs approached or even exceeded the pristine PEGs. The reason could be interpreted in two aspects: for the elevation of *T_onset-m_*, the molecular bridge of the coupling agent enhanced the bonding between the PEGs and the EG matrix; hence, more energy was required to transform the PEG crystal to liquid. As for the crystallization process, the KR-38S molecule improved the compatibility between the PEGs and the EG surface, and consequently hindered the heterogeneous nucleation of the PEGs. The reduction of Δ*T* also supported this conclusion. Therefore, it can be concluded that the modification of the EG matrix improved the *T_onset-m_*, *T_onset-c_*, and Δ*T* of the m-EPs.

As for the latent heats during phase change, the Δ*H_m_* and Δ*H_c_* of the EPs and m-EPs were lower than that of their corresponding PEGs. This was because the supporting materials in the composites couldn’t store thermal energy. Meanwhile, the Δ*H_m_* and Δ*H_c_* of the m-EPs were higher than that of the EPs. This could be attributed to the additional amount of PEGs that anchored on the m-EG matrix due to the strong chemical bonding. Furthermore, the *η_m_* and *η_c_* of the EPs were 67.38–75.83% and 71.16–80.68%, respectively, and the corresponding values for the m-EPs were 77.77–87.77% and 82.45–91.45%. It could be noted that all of the melting and crystallization enthalpies of the EPs and m-EPs were lower than the theoretical latent heat values. This was because the PEGs couldn’t be incorporated into the pore structure of the matrix, and the residual PEGs on the surface would vanish during phase transition. It was also noted that the *η_m_* values were lower than *η_c_* for both the EPs and the m-EPs, which could be ascribed to the inherent supercooling in the PEGs, as shown in the DSC curves. In conclusion, applying m-EGs as the supporting material could increase both the melting and crystallization enthalpies of the m-EPs by approximately 10%, and the enhancement on thermal storage capacity would reduce the cost of m-EPs in buildings.

#### 3.2.2. Thermal Stability

**Figure 7:**
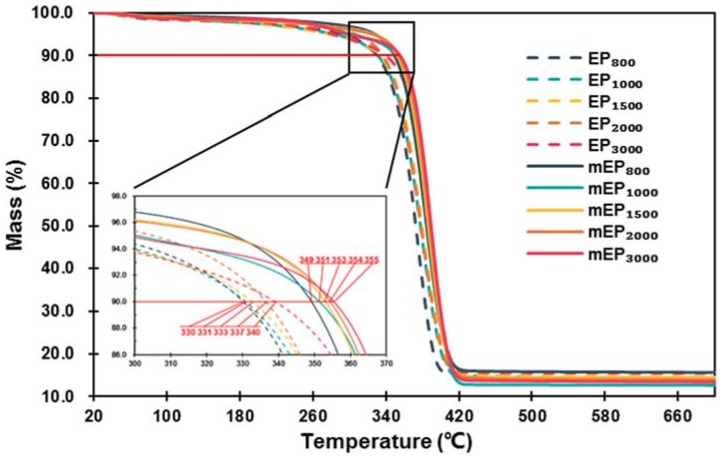
Thermogravimetric analyzer (TGA) curves of EPs and m-EPs.

The thermal stability of EPs and m-EPs was evaluated by determining the mass loss. As the TGA curves illustrated in Figure 7 indicate, the EPs and m-EPs exhibited similar thermal stability characteristics. No decomposition occurred until the heating temperatures exceeded 300 °C, and the decomposition completed at the temperatures of 420 °C. Based on the TGA curves of EPs and m-EPs, it could be inferred that the thermal stabilities of EG-based FSPCMs were satisfied at the intermediate low temperature. By comparing the initial decomposition temperatures (defined in this research as the temperature at which 10% of the weight was lost) of EPs and m-EPs, it could be noticed that the initial decomposition temperatures of the m-EPs were approximately 20 °C higher than those of the EPs. This indicated that the modification endowed the m-EPs with better thermal stability, and these kinds of composite PCMs were able to embed in building materials under high temperatures. Furthermore, it is also worth noting that the residue of the EPs and m-EPs after TGA was about 13 wt %, which was equivalent to the mass ratio of the supporting materials.

#### 3.2.3. Thermal Conductivity

**Figure 8:**
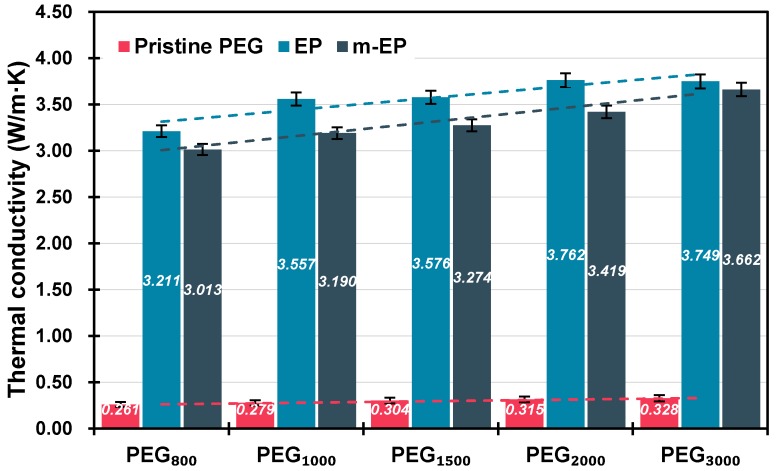
Thermal conductivity of PEGs, EGs, and m-EGs at the same packing density.

The thermal conductivities of PEGs, E Ps, and m-EPs were shown in Figure 8. Packing density affects the thermal transfer rates of EG-based PCMs significantly due to the high porosity and thermal conductivity of the EG matrix. Therefore, the EPs and m-EPs were pressed to tablet samples, and their packing densities were consistent with the pristine PEGs. As illustrated in Figure 8, the measured thermal conductivities of pristine PEGs were as low as approximately 0.3 W/m·K, which was detrimental to the thermal energy storage efficiency in the building envelopes. When the EG matrix was applied, the thermal conductivities of EPs were increased significantly to about 3.5 W/m·K. This proved the enhancement of the EG matrix on the thermal transfer rate of the latent heat thermal energy storage system. Meanwhile, the thermal conductivity of the pristine PEGs increased with the increase in their molecular mass, and a similar tendency could be detected in the thermal conductivities of the EPs and m-EPs. This result indicated that both the properties of the supporting and functional components determined the thermal performance of the FSPCMs. Furthermore, the thermal conductivity values of the m-EPs were slightly lower in comparison with those of the EPs. This might be because the modification of the organic coupling agent decreased the thermal transfer rate of the EG matrix to some extent. Even so, the thermal conductivities of the m-EPs were still 10 times higher than those of the pristine PEGs, which ensured a rapid response to the temperature fluctuations in the buildings.

## 4. Conclusions

An expanded graphite (EG)/polyethylene glycol (PEG) composite phase change material (PCM) was prepared for the purpose of bridging the gap between energy supply and demand in buildings. A titanate coupling agent KR-38S was used to build a molecular bridge between EG and PEG, and various thermal properties of the composite PCM were investigated in this paper. The following conclusions were drawn: 

The EG matrix with macropores and mesopores was beneficial to the absorption of PEG. The optimal EG particle size for PEG absorption was determined as 125 μm, and the maximum mass ratio of PEG to EG was 1:7 in this research. The modification of KR-38S increased the quantity of the oxygen functional groups on the EG matrix, and a stable molecular bridge was established between the EG matrix and PEG. The optimal modification condition was obtained by adding 3 wt % of KR-38S at 60 °C. Compared with the EPs, the melting and crystallization temperatures of the m-EPs showed little variation, but the phase change temperature ranges and supercooling degree decreased significantly, indicating that the phase transition abilities of the EPs were enhanced after modification. The melting and crystallization enthalpies of the m-EPs increased by approximately 10%, and the initial decomposition temperatures rose by about 20 °C in comparison with the EPs. These results indicated the improvements to the thermal energy storage efficiency and stability of the m-EPs, which are beneficial for the application of m-EPs in building envelopes. The thermal conductivities of the m-EPs were 10 times higher than those of the pristine PEGs. This result indicated that using m-EPs in building envelopes could enhance the heat transfer rate as well as ensure a rapid response to temperature fluctuations.

In summary, using titanate coupling agent could build a molecular bridge in m-EPs, and improve the phase change temperature range, supercooling, heat storage density, thermal stability, and thermal conductivity of m-EPs. This research can benefit the application of PCMs in building energy conservation. 

## Figures and Tables

**Table 1:** Basic properties of the polyethylene glycols (PEGs) used in this research.

Categories	*Mr*	*T_onset-m_* (°C)	*T_onset-c_* (°C)	Δ*T* (°C)	Δ*H_m_* (J/g)	Δ*H_c_* (J/g)	*λ* (W/m·K)
PEG_800_	800	21.79	23.56	−1.77	143.51	126.63	0.2606
PEG_1000_	1000	27.92	30.74	−2.82	163.10	152.08	0.2790
PEG_1500_	1500	43.36	31.04	12.32	170.34	159.24	0.3038
PEG_2000_	2000	50.38	40.76	9.62	187.24	171.87	0.3151
PEG_3000_	3000	55.15	43.24	11.91	186.50	160.86	0.3281

Note: *Mr*: relative molecular mass; *T_onset-m_*: onset melting temperature; *T_onset-c_*: onset crystallization temperature; Δ*T*: supercooling degree, equals the temperature difference between *T_onset-m_* and *T_onset-c_*; Δ*H_m_*: melting enthalpy; Δ*H_c_*: crystallization enthalpy; *λ*: thermal conductivity.

**Table 2:** Basic properties of expanded graphites (EGs) used in this research.

Categories	Average Particle Size (μm)	Specific Surface Area (m^2^/g)	Pore Volume (cm^3^/g)	Average Pore Radius (μm)
EG_45_	45	52.1099	1.4110	0.2101
EG_75_	75	45.7174	3.2946	0.2584
EG_125_	125	41.5314	7.1265	0.3152
EG_180_	180	40.1269	7.7437	0.3273
EG_300_	300	32.9647	9.2215	0.3439

**Table 3:** Pore structure parameters of EGs with the maximum absorptive capacities.

Categories	Specific Surface Area (m^2^/g)	Pore Volume (cm^3^/g)	Average Pore Radius (μm)	Maximum Mass Ratio of EG to PEG
EG_45_	21.5468	0.0954	0.0109	1:4
EG_75_	24.3368	0.1089	0.0142	1:4
EG_125_	17.6056	0.1354	0.0379	1:7
EG_180_	15.3005	0.2025	0.0326	1:7
EG_300_	14.5487	0.2944	0.0335	1:8

**Table 4:** Micromorphologies of EGs before and after PEG absorption.

Categories	Before Absorption	After Absorption
EG_45_	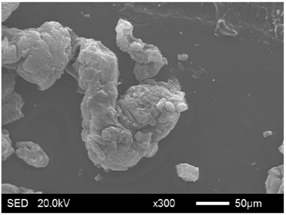	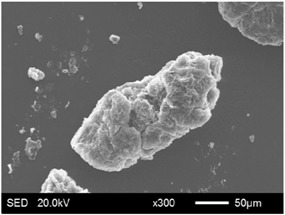
EG_75_	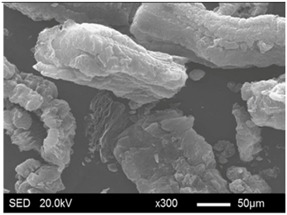	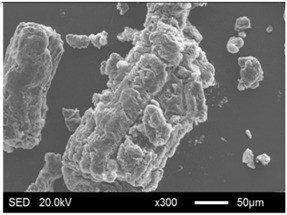
EG_125_	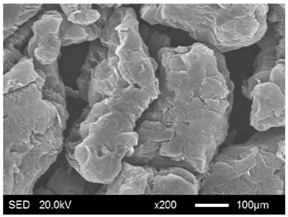	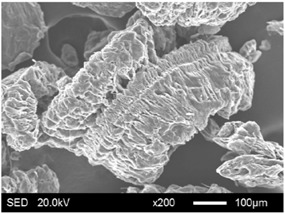
EG_180_	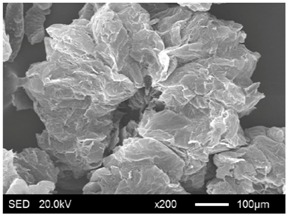	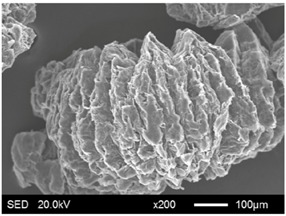
EG_300_	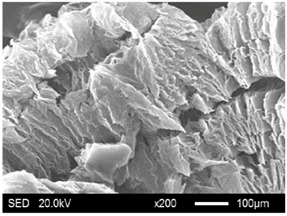	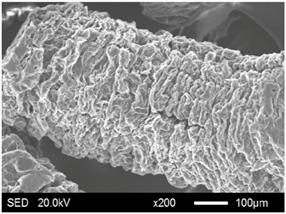

**Table 5:** Thermal properties of EPs and m-EPs.

Categories	*T_onset-m_* (°C)	*T_onset-c_* (°C)	Δ*T* (°C)	Δ*H_m_* (J/g)	Δ*H_c_* (J/g)	*η_m_* (%)	*η_c_* (%)
EP_800_	14.30	17.53	−3.23	89.50	80.61	71.27	72.75
EP_1000_	23.96	18.49	5.47	108.22	101.92	75.83	76.59
EP_1500_	37.67	22.36	15.31	104.13	104.56	69.86	75.04
EP_2000_	39.29	26.36	12.93	110.40	107.01	67.38	71.16
EP_3000_	45.98	30.63	15.35	120.50	113.56	73.84	80.68
m-EP_800_	22.64	21.90	0.74	97.66	92.91	77.77	83.85
m-EP_1000_	30.18	33.64	−3.46	121.89	116.80	85.41	87.77
m-EP_1500_	42.67	33.93	8.74	122.94	114.88	82.48	82.45
m-EP_2000_	51.95	41.66	10.29	143.79	137.53	87.77	91.45
m-EP_3000_	54.63	41.27	13.36	141.60	124.12	86.77	88.18

Note: *η_m_:* melting enthalpy efficiency, $\eta_{m}=\;\frac{{\Delta H}_{m}\;\mathrm{of}\;\mathrm{EP}\;\mathrm{or}\;m-\mathrm{EP}}{\;\left({\Delta H}_{m}\;\mathrm{of}\;\mathrm{PEG}\right)\;\times \;\left(\mathrm{PEG}\;\%\;\mathrm{in}\;\mathrm{EP}\;\mathrm{or}\;m-\mathrm{EP}\right)\;}\times \;100$%; *η_c_*: crystallization enthalpy efficiency, $\eta_{c}=\frac{{\Delta H}_{c}\;\mathrm{of}\;\mathrm{EP}\;\mathrm{or}\;m-\mathrm{EP}}{\left({\Delta H}_{c}\;\mathrm{of}\;\mathrm{PEG}\right)\;\times \;\left(\mathrm{PEG}\;\%\;\mathrm{in}\;\mathrm{EP}\;\mathrm{or}\;m-\mathrm{EP}\right)\;}\;\times \;100$%.
